# Intron Retention as a Homeostatic State Variable for Drug Response and Recovery: Lessons from Depression for Broader Applications

**DOI:** 10.3390/ijms27083539

**Published:** 2026-04-16

**Authors:** Norihiro Okada, Kenshiro Oshima, Akiko Maruko, Akinori Nishi, Yoshinori Kobayashi

**Affiliations:** 1 School of Pharmacy, Kitasato University, Tokyo 108-8641, Japan; 2 TSUMURA Advanced Technology Research Laboratories, Research & Development Division, TSUMURA & Co., Ibaraki 300-1192, Japan; 3 Oriental Medicine Research Center, School of Pharmacy, Kitasato University, Tokyo 108-8641, Japan

**Keywords:** intron retention, homeostasis, pharmacodynamic biomarker, depression, drug response, inflammation, mild cognitive impairment

## Abstract

Clinically robust molecular biomarkers for depression have remained elusive, despite extensive transcriptomic research. This gap is consequential: depression is prevalent and heterogeneous, yet objective measures to quantify burden, stratify patients, and track recovery remain limited. Here, we review evidence that intron retention (IR) can serve as a homeostatic state variable—and therefore a sensitive biomarker—reporting stress adaptation and recovery at an upstream regulatory layer, often preceding or outperforming differential gene expression (DEG) readouts. Mechanistically, IR enables bidirectional fine-tuning of effective gene output: increased IR (IncIR) can throttle output under overload, whereas decreased IR (DecIR) releases this brake to restore gene output. Because these shifts are reversible and treatment-responsive, IR signatures can function not only as disease markers but also as pharmacodynamic metrics for blood-based monitoring of drug response and recovery. To evaluate the clinical utility of IR, we use depression as a proof of concept and focus on two interventions: (i) the Kampo formula hangekobokuto (HKT), which is associated with IR normalization consistent with reduced peripheral inflammatory load; and (ii) ketamine, where IR patterns measured before ketamine treatment in non-responders are linked to stronger innate-immune/antiviral activity, suggesting a higher inflammatory load that may limit treatment benefit. Finally, we discuss transdiagnostic extensions beyond depression, using early cognitive decline (mild cognitive impairment, MCI) as a stringent, biologically distal test case for blood-based IR/DI readouts and motivating independent cohort replication and longitudinal validation.

## 1. Introduction: The Unmet Need for Actionable Biomarkers of Depression

Major depressive disorder (MDD) remains among the most disabling illnesses worldwide, yet clinical decision-making still relies predominantly on symptom-based assessments and trial-and-error treatment selection. A central bottleneck is the lack of actionable molecular biomarkers that can (i) quantify disease burden in an individual, (ii) stratify biologically distinct subtypes, and (iii) track recovery trajectories during treatment. This gap persists despite decades of transcriptomic, proteomic, inflammatory, and imaging studies, suggesting that the prevailing biomarker paradigm may be targeting the wrong molecular “layer” [1,2].

Blood transcriptomics is attractive because it is scalable and minimally invasive and because peripheral immune/inflammatory states plausibly contribute to depressive symptoms in at least a subset of patients [3,4,5,6,7,8,9]. However, the dominant approach—differential gene expression (DGE)—often yields signatures that are cohort-fragile, sensitive to sampling context, and difficult to transfer across studies [10,11,12]. Whole blood is a heterogeneous mixture of immune subsets whose proportions vary across individuals and time; lifestyle, circadian timing, subclinical infection, comorbidities, and medication history further shape steady-state mRNA abundance. Even when statistically significant, DGE frequently represents downstream outputs: the end products of multiple regulatory steps rather than the upstream control settings that govern adaptation and recovery [1,13,14,15].

**Figure 1:**
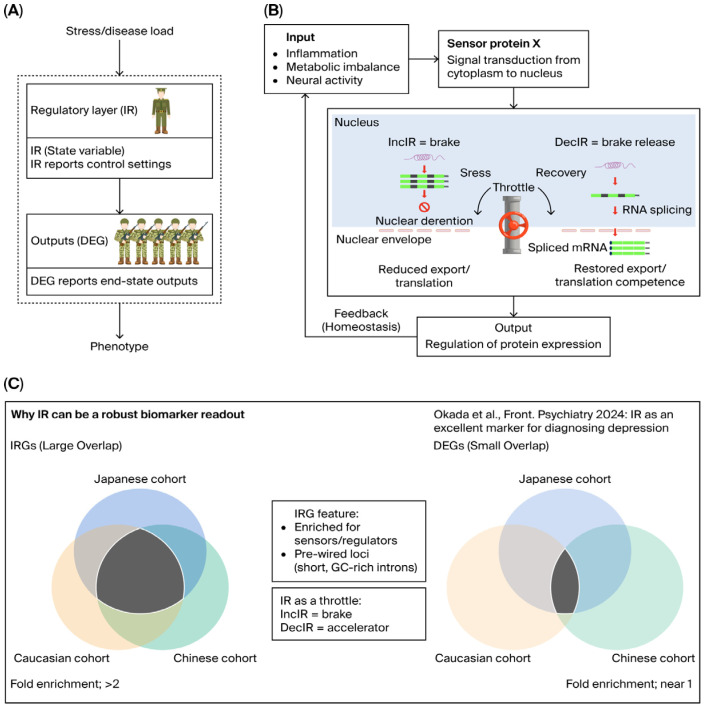
Conceptual framework for intron retention (IR) as a homeostatic state variable for pharmacodynamic monitoring and its cross-cohort robustness relative to DEG outputs. (**A**) Positioning of IR relative to conventional differential gene expression (DEG): IR reports an upstream RNA-processing layer that shapes effective gene output like a commander, whereas DEGs represent downstream abundance outputs that are often more context- and composition-sensitive in whole blood like soldiers. (**B**) The “throttle/IR-Homeostat” model: increased intron retention (IncIR) acts as a reversible brake on effective gene output by reducing the pool of mature export- and translation-competent mRNAs, while decreased intron retention (DecIR) releases this brake to facilitate recovery of gene output. (**C**) Cross-cohort reproducibility of IR programs: Venn diagram showing overlap of IR genes defined as IncIR + DecIR across three independent depression blood RNA-seq cohorts (this study and two external cohorts), with fold enrichment (observed/expected overlap) and Fisher’s exact test *p*-values (left). Corresponding cross-cohort overlap analysis for DEGs (up + down) across the same cohorts, showing markedly weaker or depleted overlap relative to expectation (right). Source/license: panels (**A**,**B**) were newly created for this review. Panel (**C**) is adapted from Figure 8D,E in [20]), used under the Creative Commons Attribution (CC BY 4.0) license.

These considerations motivate a shift from “downstream abundance endpoints” to upstream state variables—molecular readouts that reflect upstream regulatory mechanisms and therefore generalize better across cohorts while remaining sensitive to within-individual changes. In this review, we focus on intron retention (IR) and argue that it can serve as a homeostatic state variable—and therefore a sensitive biomarker—reporting stress adaptation and recovery at an upstream regulatory layer, often preceding or outperforming conventional DGE readouts (Figure 1A) [1]. By “homeostatic state variable,” we mean a molecular readout that reflects the upstream regulatory state of a biological system under stress and during recovery, rather than merely its downstream abundance output. In this sense, IR can be viewed as a post-transcriptional “throttle” on effective gene output: it is informative not simply because it changes in disease but because it reports how cells allocate RNA-processing capacity under physiological load and how this allocation is reset as recovery proceeds. We further emphasize that this property makes IR not only a disease marker but also a pharmacodynamic instrument for evaluating treatment efficacy and recovery kinetics [16,17,18,19].

Our recent IJMS review [1] proposed the “IR-Homeostat” hypothesis: intron retention/detained intron (IR/DI) switching is an evolutionarily conserved, switchable fine-tuning layer that couples homeostatic inputs to tunable gene-output states. That article focused on the mechanistic and evolutionary rationale—why IR is well positioned to implement feedback-like control of effective gene output across diverse stressors.

**Figure 2:**
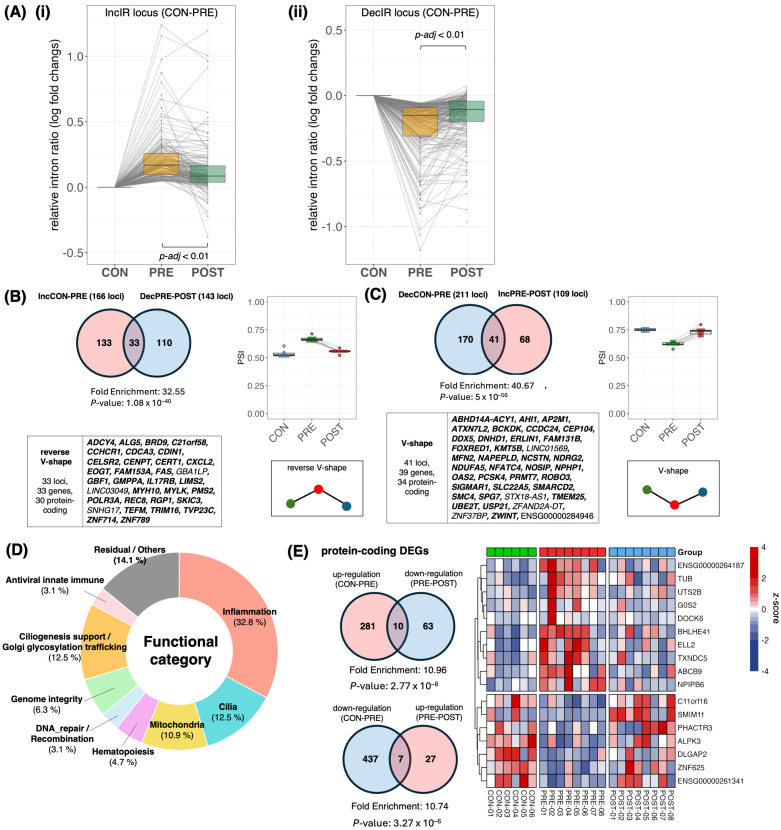
The Kampo formula hangekobokuto (HKT) normalizes intron-retention programs in peripheral blood and defines directional IR recovery motifs. (**A**) Global recovery trends across significant IR loci. Loci showing increased intron retention in patients before treatment relative to controls (IncIR; CON→PRE) (**i**) and loci showing decreased intron retention (DecIR; CON→PRE) (**ii**) are tracked across CON, PRE, and POST. Grey lines indicate individual loci; boxplots summarize distributions of relative intron-ratio changes. (**B**) Reverse V-shaped recovery loci. Venn diagram showing overlap between IncIR loci (CON→PRE) and loci that show decreased IR after treatment (PRE→POST); the overlap defines reverse V-shaped recovery loci. Gene symbols are listed, and a representative PSI summary across CON/PRE/POST with a schematic motif is shown. (**C**) V-shaped recovery loci. Venn diagram showing overlap between DecIR loci (CON→PRE) and loci that show increased IR after treatment (PRE→POST); the overlap defines V-shaped recovery loci. Gene list, PSI summary, and motif schematic are shown as in (**B**). (**D**) Functional breakdown of recovered protein-coding IR genes (*n* = 64; 30 from reverse V-shape + 34 from V-shape), categorized by literature-based annotation (DNA repair/recombination, hematopoiesis, mitochondria, cilia, inflammation, and others). Table S1; updated annotation of genes previously grouped as “Others” (*n* = 23), partitioned into genome integrity, ciliogenesis-support (Golgi/glycosylation/trafficking), antiviral/innate-immune, and residual categories (percentages shown within the “Others” subset). (**E**) Comparison with differential gene expression (DEG) recovery. Venn diagrams identify protein-coding DEGs exhibiting reciprocal “recovery” patterns across CON/PRE/POST, and a heatmap shows z-scored expression of representative recovery DEGs across samples/groups. Where indicated, fold enrichment was calculated as the observed overlap divided by the expected overlap under independence (|A| × |B|/N), using the total number of evaluated loci/genes (N) in the corresponding analysis as background; *p*-values were assessed by Fisher’s exact test. Abbreviations: CON, controls; PRE, before medication/treatment (pre-treatment); POST, after medication/treatment (post-treatment); IR, intron retention; IncIR, increased intron retention; DecIR, decreased intron retention; PSI, percent spliced in; DEG, differentially expressed gene. Source/license: adapted from Figure 6 in [20], used under the Creative Commons Attribution (CC BY 4.0) license.

Here, we write a complementary, application-centered review. Rather than revisiting the full conceptual foundation, we focus on how IR can be used as a practical pharmacodynamic readout to quantify drug response and recovery in vivo. To make this argument concrete, we build the main narrative around two complementary clinical datasets. First, a Kampo medicine intervention (hangekobokuto; HKT) demonstrates drug-responsive normalization of IR programs (Figure 2, from Figure 6 in [20]). Second, ketamine response heterogeneity shows how IR can provide a molecular characterization of clinically defined responders versus non-responders and generate mechanistic hypotheses for non-response (See the later section [21]). Finally, we address outliers, arguing that they often reflect clinically meaningful heterogeneity rather than noise. Notably, the impulse to treat outliers as artifacts is largely inherited from DGE-based workflows; IR-based analyses do not require this premise and can incorporate biological extremes as part of the signal (Section 5) [20,21]. We close by briefly extending this pharmacodynamic logic beyond depression, highlighting early cognitive decline (MCI) as a setting where baseline case–control IR separation may be modest but within-individual IR trajectories could still provide a sensitive readout of treatment engagement and recovery.

## 2. Conceptual Framework: IR as an Upstream “Throttle” on Effective Gene Output

Intron retention (IR) occurs when an intron remains within a transcript that would otherwise be fully spliced. Retained (or detained) introns can influence transcript fate through nuclear retention/detention, altered export, changes in translation competence, or decay routes, depending on locus architecture and cellular context. Importantly, multiple systems indicate that IR can be regulated, switchable, and reversible, rather than merely a splicing error [1,19,22,23].

A useful translational abstraction is to treat IR as a post-transcriptional throttle controlling effective gene output—the supply of mature, export-competent mRNAs that can be translated. Within this throttle model (Figure 1B), IncIR (increased intron retention) functions as a reversible brake that dampens effective output when the system experiences overload or when energetic and proteostatic capacity is constrained; DecIR (decreased intron retention) releases this brake, enabling rapid restoration of output during recovery. This “brake/accelerator” logic aligns naturally with a homeostatic view of regulation: cells must prevent overshoot, allocate limited processing capacity, and coordinate recovery programs without requiring complete transcriptional rewiring [18,24].

This conceptual layer matters for biomarker design. Differential gene expression (DGE) is often an endpoint readout that reflects the combined effects of many upstream decisions as a net abundance change. By contrast, IR reports an upstream RNA-processing layer that can change earlier and may generalize better because it is tied to regulated processing decisions. This premise is consistent with the broader “IR-Homeostat” concept discussed in our recent IJMS review [1], in which IR/DI switching is treated as a conserved regulatory layer that links homeostatic inputs to tunable gene-output states [18,25,26,27,28].

An empirical hallmark of an upstream state variable is cross-cohort reproducibility (Figure 1C). In our depression blood analyses [20], IR-defined programs (IncIR + DecIR) showed strong overlap across independent cohorts, including a Japanese cohort, a Caucasian cohort (Cathomas et al. [29]), and a Chinese cohort (Zhang et al. [30]), with overlaps enriched well above random expectation (fold enrichment ~2.5–3.2; Figure 1C (left), adapted from Figure 8D in [20]). In contrast, the corresponding differentially expressed gene (DEG) sets (up + down) exhibited weak or even depleted overlap across the same cohorts (fold enrichment ~0.6–0.8), yielding only a minimal shared core (Figure 1C (right), adapted from Figure 8D,E in [20]). This contrast is consistent with IR capturing a more conserved, upstream RNA-processing layer, whereas DEGs more often reflect downstream, context- and composition-sensitive outputs in whole blood.

Using this conceptual framework, we next examine two intervention-based proof-of-concept datasets to illustrate how IR can function as a pharmacodynamic readout of recovery and a molecular lens for interpreting response heterogeneity.

## 3. Case Study 1: Hangekobokuto Demonstrates Drug-Responsive Normalization of IR Programs

A decisive test for any candidate biomarker is whether it behaves as a pharmacodynamic readout, i.e., whether it not only distinguishes states but also moves in the correct direction with effective treatment. In our previous study [20], we analyzed RNA-seq data from PBMC in controls and subjects sampled before and after administration of the Kampo formula hangekobokuto (HKT) and mapped IR changes across conditions.

### 3.1. Recovery Patterns Reveal “V-Shape” and “Reverse-V-Shape” IR Loci

Figure 2A(i,ii) (from Figure 6A in [20]) highlight two reciprocal recovery trends, respectively: loci that show increased intron retention in subjects before treatment relative to controls (IncIR; CON→PRE) tend to shift back toward control-like levels after treatment (PRE→POST), whereas loci that display decreased intron retention before treatment (DecIR; CON→PRE) tend to increase toward control-like levels after treatment (PRE→POST). Importantly, Figure 2B,C (from Figure 6B,C in [20]) formalize these trends as two interpretable recovery motifs:

- Loci with reverse V-shape: Loci that increase in IR before treatment (vs. control) and decrease after treatment (IncIR→recovery).
- Loci with V-shape: Loci that decrease in IR before treatment and increase after treatment (DecIR→recovery).

These patterns are not merely descriptive; they provide a practical way to define treatment-responsive IR loci that encode the directionality of homeostatic restoration. In a pharmacodynamic framework, such loci serve as quantitative “dials” for recovery.

### 3.2. The Recovered IR Program Is Enriched for Inflammation-Linked Biology

A striking aspect of Figure 2D is the functional composition of the IR loci that normalize with HKT [20]. The recovered set shows strong representation of inflammation/immune-related categories, alongside cilia, mitochondria, hematopoiesis, and DNA repair/recombination, and the inset further resolves the former “Others” into genome integrity, ciliogenesis-support (Golgi/glycosylation/trafficking), and antiviral/innate-immune themes. This supports a coherent mechanistic narrative: HKT improves symptoms and the molecular state, plausibly by dampening peripheral inflammatory/innate-immune activation, and IR captures the associated upstream shift in regulated RNA-processing states. This point is particularly valuable for a feature review aimed at drug response and recovery monitoring. Rather than asserting a single pathway, the IR readout allows one to frame recovery as multi-layer normalization with a clearly visible inflammatory axis. This observation naturally raises the next question: do these IR recovery signatures align with physiological anti-inflammatory and innate-immune-modulating effects previously reported for HKT/BHT in independent experimental systems?

### 3.3. Convergent Evidence: IR Recovery Aligns with Physiological Normalization Reported for HKT/BHT

A key question is whether the IR recovery motifs observed after HKT reflect a biologically meaningful restoration of physiological state. Although our dataset does not directly quantify all inflammatory mediators, multiple independent studies report that HKT/BHT dampens inflammatory and innate-immune activation through aspects such as reduced iNOS/NO signaling [31], decreased pro-inflammatory cytokines [32], and suppression of inflammasome-related pathways [33]. Notably, the recovered IR genes include nodes that map onto these axes (e.g., NOSIP for the NO axis, CXCL2 for leukocyte recruitment, IL17RB for IL-17-related inflammatory tone, and OAS2 for innate antiviral programs), and re-annotation of the former “Others” in Figure 6D in [20] further highlights endomembrane/glycosylation themes consistent with inflammatory regulation. Table 1 summarizes representative mechanistic reports and their correspondence to recovered IR genes. Notably, ~18% (13/72) of the recovered IR genes functionally map to innate-immune/inflammatory or NO-related axes, supporting cross-study concordance with reported HKT/BHT-mediated dampening of inflammatory and innate-immune activation. Taken together, these cross-study concordances provide convergent support for the view that IR normalization can serve as a physiologically informative pharmacodynamic readout of recovery, while encouraging future prospective studies that measure IR alongside physiological markers in the same cohort to assess how closely they track and whether they predict outcomes [31,32,33,34,35,36].

### 3.4. IR Outperforms DGE as a Recovery Readout in the Same Dataset (With Fold-Enrichment Quantification)

Figure 2E shows recovery signals captured by conventional differentially expressed genes (DEGs), which can be compared with those captured by intron retention (IR) (Figure 2B,C) in the same dataset. IR defines sizable, directionally interpretable recovery sets (V-shape and reverse V-shape), whereas the analogous DEG “recovery” sets (up in disease and down after treatment, or the reverse) are comparatively small and show limited concordance. To quantify this contrast beyond visual inspection, we report fold enrichment (observed/expected overlap) for IR- and DEG-defined recovery sets using the expressed gene universe in this dataset as background, with significance assessed by Fisher’s exact test (Figure 2E). Across recovery definitions, IR overlaps show markedly stronger enrichment (fold enrichment 32.55–40.67) than DEG overlaps (fold enrichment 10.74–10.96), indicating that IR captures a more coherent recovery program within the same sampling and intervention framework [20].

Importantly, the recovered IR gene set and the recovered DEG set show little to no direct gene-level overlap, which is consistent with a layered interpretation rather than a contradiction: IR can modulate effective gene output through RNA-processing decisions without requiring a large net change in steady-state mRNA abundance, while DEGs preferentially report downstream, context- and composition-sensitive outputs in whole blood (Figure 1A). This dataset-level contrast motivates the next question addressed below—how IR-defined state nodes and DEG-defined output genes are coupled within shared biological axes.

### 3.5. Network Coupling Between IR-Defined State Nodes and DEG-Defined Outputs (Cytoscape/STRING)

**Figure 3:**
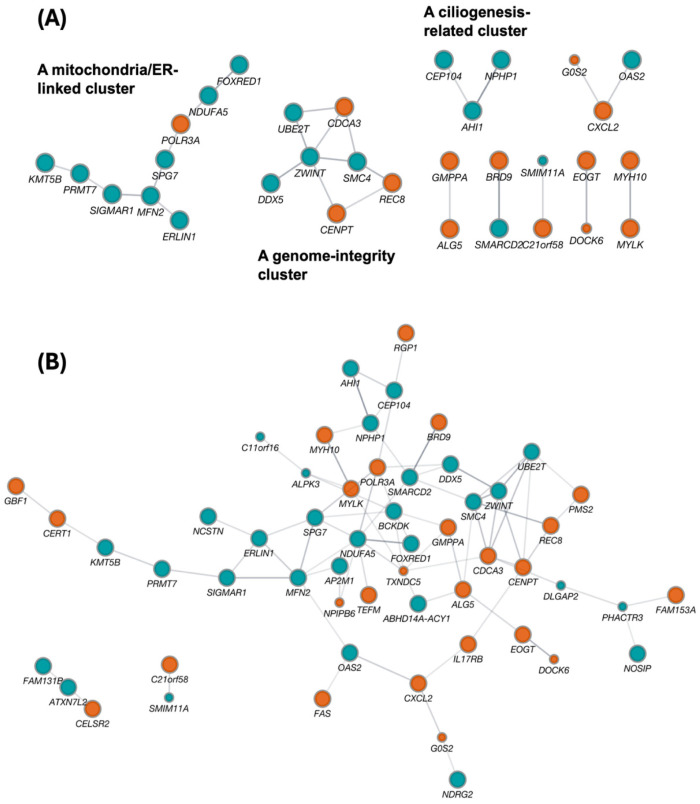
Cytoscape/STRING network linking recovered IR genes and recovered DEGs. Protein–protein interaction (PPI) networks were constructed in Cytoscape using STRING (*Homo sapiens*) from the union of recovered IR genes (*n* = 64, protein-coding recovery IRGs from Figure 2B,C) and recovered DEGs (*n* = 17, recovery DEGs from Figure 2E). Node size indicates gene class (large nodes, recovered IRGs; small nodes, recovered DEGs). Node color denotes the recovery motif (reverse V-shape, red circle; V-shape, blue circle, as defined in Figure 2B,C,E). Edges indicate STRING functional associations (combined score threshold as specified). (**A**) Network at combined score ≥ 0.4 (primary, medium-confidence view). (**B**) Network at combined score ≥ 0.2 (expanded, low-confidence view) shown for exploratory context; interactions unique to (**B**) should be interpreted as hypothesis-generating. Isolated nodes are displayed to indicate recovered genes without STRING-supported connections at the corresponding threshold. Source/license: panels (**A**,**B**) were newly created for this review. Abbreviations: IRG, intron-retention gene; DEG, differentially expressed gene; PPI, protein–protein interaction; STRING, Search Tool for the Retrieval of Interacting Genes/Proteins.

To visualize potential bridges between IR-defined state nodes and DEG-defined output genes, we constructed a protein–protein interaction network in Cytoscape (v.3.10.4) using STRING (v.2.2.0), combining recovered IR genes (*n* = 64) and recovered DEGs (*n* = 17) (Figure 3). At medium confidence (STRING combined score ≥ 0.4; Figure 3A), recovered IR genes form coherent multi-gene modules that recapitulate major recovery axes highlighted elsewhere in this review, including (i) a mitochondria/ER-linked cluster (e.g., MFN2–ERLIN1–SPG7 with POLR3A–NDUFA5–FOXRED1) [37,38,39], (ii) a genome-integrity cluster (e.g., ZWINT–SMC4–CENPT/REC8 with UBE2T/DDX5) [40,41,42,43], and (iii) a ciliogenesis-related cluster (e.g., AHI1–CEP104–NPHP1) [44,45,46]. In contrast, most recovered DEGs appear peripheral or disconnected at this threshold, consistent with their role as downstream, more context-dependent outputs rather than shared upstream state-control nodes [47,48].

Notably, a small number of links between the IR-defined state layer and the DEG-defined output layer remain detectable even at a STRING combined score ≥ 0.4, providing concrete examples of axis-level coupling. For example, the immune/innate axis is captured by a CXCL2-centered neighborhood that links the recovered DEG G0S2 to two IR-recovered nodes, CXCL2 and OAS2. Whereas CXCL2 and OAS2 mark chemokine/inflammatory and interferon-inducible antiviral components of this IR-defined state module, respectively, G0S2 is better interpreted here as a downstream output associated with the same broader inflammatory context. This pattern is consistent with coupling between chemokine-linked inflammatory signaling and interferon-inducible antiviral programs [49,50] and aligns with the “immune/innate” correspondence summarized in our DEG–IR mapping table (Table 2). Similarly, a cytoskeletal/adhesion output gene, DOCK6, connects to the IR-recovered node EOGT within the same broader axis [51,52]. In this pairing, DOCK6 points more directly to cytoskeletal remodeling, whereas EOGT suggests an upstream glycosylation-related regulatory context. These bridges support the view that the IR-defined state layer and the DEG-defined output layer may be coupled through shared pathways even when direct gene overlap is minimal.

To make this correspondence explicit, Table 2 provides a compact DEG–IR cross-walk derived from the STRING/Cytoscape network by combining conservative direct links seen at the medium-confidence threshold (Figure 3A) with broader axis-level relatedness visible in the expanded network view (Figure 3B). Recovered DEGs that retained direct DEG–IR connections at a STRING combined score ≥ 0.4 were treated as concrete examples of coupling between the IR-defined state layer and the DEG-defined output layer. For recovered DEGs without a stable direct IR link at this threshold, assignment to an IR-centered recovery axis was based on their local network neighborhood and shared biological theme in the expanded network view. Representative IR nodes were then listed for each assigned axis. Relationships supported only in Figure 3B were interpreted as exploratory and hypothesis-generating rather than as definitive evidence of DEG–IR coupling.

When the STRING threshold is relaxed (combined score ≥ 0.2; Figure 3B), the network expands into broader functional neighborhoods and reveals additional candidate bridges between IR-defined modules and DEG-defined outputs. We do not treat these lower-confidence edges as evidence of concrete DEG–IR coupling. Rather, Figure 3B is intended as an exploratory view of axis-level relatedness, showing how IR-centered state modules and DEG outputs may converge within broader biological neighborhoods even when stable direct links are not retained at the more conservative threshold in Figure 3A. In practical terms, of the 17 recovered DEGs carried forward from Figure 2E and Figure 3A provides conservative placement for three, whereas Figure 3B suggests broader axis-level positioning for nine DEG outputs. In this sense, Figure 3B complements Figure 3A by visualizing potential continuity between the state layer and the output layer and by helping prioritize mechanistic follow-up. Overall, the network analysis complements the fold-enrichment results by providing a structural explanation for a layered architecture: IR changes preferentially mark upstream state-control modules, whereas DEGs report selective downstream outputs, with limited direct overlap but detectable convergence within shared biological axes [1].

This layered view also fits well with the “commander–soldier” analogy we discussed in our recent Research Square preprint [53] (see also Figure 1A), in which IRGs behave as multi-process “command-tier” nodes while DEGs more often reflect task-specific downstream outputs. In that study, a Cytoscape/STRING network highlighted the IRG *Ucp2* as a layer-bridging node positioned upstream of multiple downstream DEG neighborhoods (Figure 6 in that paper), consistent with an IR-defined homeostatic regulator linking to diverse output routes. This emphasis on Ucp2 is biologically plausible: *UCP2* is required for sustained efferocytosis by phagocytes, enabling continued clearance of apoptotic cells and limiting inflammatory spillover that could perturb immune-educational microenvironments such as the thymus [54,55,56]. Accordingly, an IR event on *Ucp2* represents a plausible post-transcriptional throttle on efferocytosis-linked immune homeostasis. Complementarily, a gene-concept network (cnetplot) showed that IRGs exhibit higher functional connectivity than DEGs—IRGs more frequently link to multiple GO terms, whereas DEGs tend to occupy more term-restricted neighborhoods (Figure 7 in that paper, e.g., 51.1% vs. 27.6% multi-term overlap in our analysis). Together with the present depression recovery network, these observations support a general architectural principle: IR preferentially marks upstream “command-tier” state nodes, whereas DEGs report more selective, context-dependent downstream programs [1].

## 4. Case Study 2: Ketamine Links Non-Response to an Innate-Immune/Antiviral-Load State in Depression

**Figure 4:**
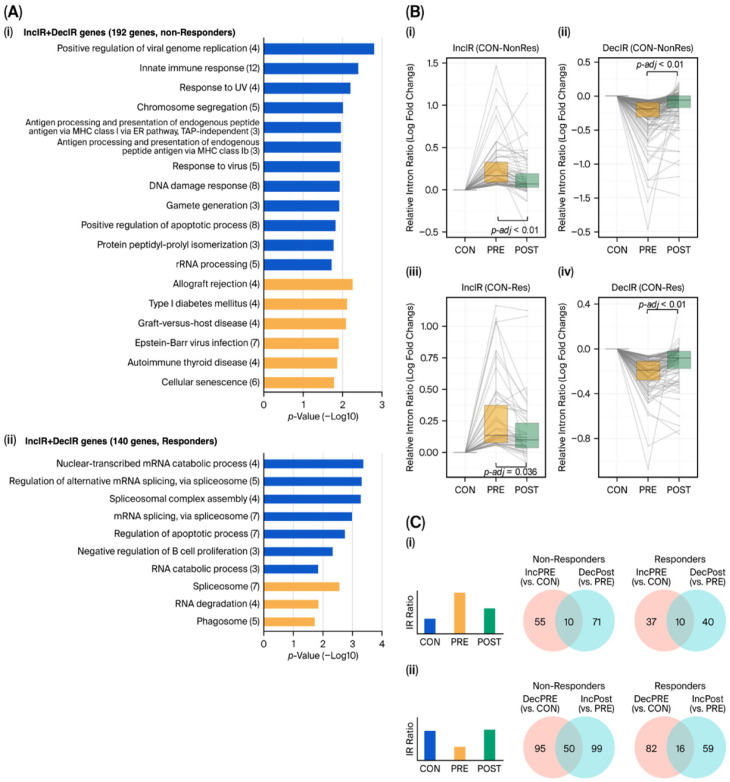

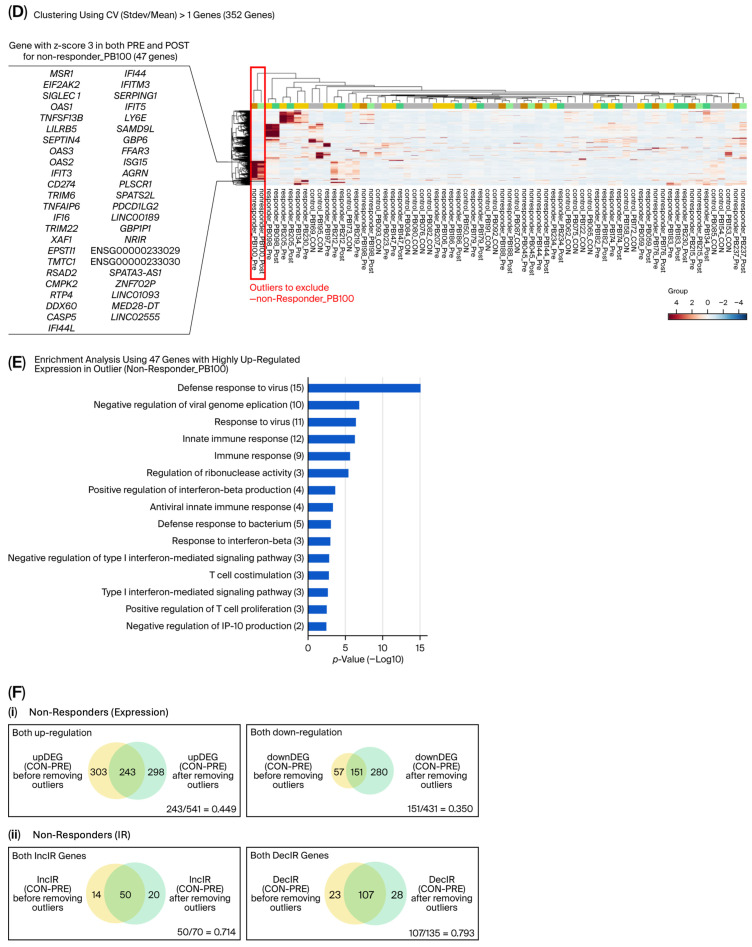
Ketamine dataset: IR pathway enrichment, IR recovery dynamics, recovery-motif definitions, and outlier sensitivity of expression versus IR readouts. (**A**) GO Biological Process and KEGG enrichment of merged IncIR + DecIR genes at PRE vs. CON for clinically defined non-responders (**i**) and responders (**ii**); bars indicate −log10 (*p*-value) (GO in blue, KEGG in orange). (**B**) Box-and-whisker plots showing distributions of relative intron-ratio changes for IncIR loci (CON→PRE) (**i**,**iii**) and DecIR loci (CON→PRE) (**ii**,**iv**) across CON, PRE, and POST, shown separately for non-responders (**i**,**ii**) and responders (**iii**,**iv**); lines indicate individual loci and adjusted *p*-values are indicated. (**C**) Venn diagrams defining recovery motifs in each clinical group: reverse V-shaped recovery (IncPRE vs. CON intersecting DecPOST vs. PRE) (**i**) and V-shaped recovery (DecPRE vs. CON intersecting IncPOST vs. PRE) with counts shown (**ii**). (**D**) Expression heatmap across individuals highlighting a non-responder outlier sample (PB100; labeled “non-Responder_PB100”) with 47 genes showing a z-score > 3 in both PRE and POST (gene list shown). (**E**) Left, enrichment analysis of the 47 PB100 up-regulated genes. Right, Venn diagrams comparing overlaps before and after excluding PB100 for expression-based DEGs and IR-based gene sets. (**F**) Venn diagram comparing DEGs or IRGs before and after excluding the outlier in the non-responder group. (**i**) Comparison of DEGs that were both upregulated on the right and downregulated on the left and the proportions of genes that remained stable after outlier exclusion are shown in the lower panel. (**ii**) Comparison of IRGs that were both IncIR genes on the right and both DecIR genes on the left, and the proportions of genes that remained stable after outlier exclusion are shown on in the lower panel. Source/license: schematic redrawn by the authors based on concepts described in [21]; no original artwork was reproduced. Abbreviations: CON, healthy controls; PRE, pre-treatment (before ketamine); POST, post-treatment (after ketamine); IR, intron retention; IncIR, increased intron retention; DecIR, decreased intron retention; GO, Gene Ontology; DEG, differentially expressed gene.

Ketamine provides a complementary proving ground because it highlights two clinically central realities: (i) some patients respond rapidly and (ii) non-response is common. In our analysis, non-responders showed a heightened innate-immune/antiviral signature. Using whole-blood RNA-seq in treatment-resistant depression, we asked whether IR can (a) characterize clinically defined responders versus non-responders at the pre-treatment (PRE) state and (b) provide a pharmacodynamic readout that remains informative under real-world biological variability (Figure 4) [21].

### 4.1. Pre-Treatment IR Programs Reveal an Innate-Immune/Viral-Load State in Non-Responders (Figure 4A)

Figure 4A summarizes the pathway enrichment of IR-altered genes (merged IncIR + DecIR) at PRE vs. CON in clinically defined non-responders and responders. In non-responders, the top enriched terms prominently include viral/innate-immune and interferon-related biology, whereas responders show a distinct enrichment profile more consistent with RNA processing/splicing and related homeostatic programs. This contrast shifts the framing from “which genes differ from controls?” to “what pre-treatment state might constrain recovery capacity?”

### 4.2. IR Shows Pharmacodynamic Engagement in Both Groups (Figure 4B)

Importantly, treatment engagement at the IR layer is not confined to responders. In both clinically defined groups, loci classified as IncIR at PRE vs. CON tend to move toward control-like levels at POST (Figure 4B(i,iii)), and loci classified as DecIR at PRE vs. CON show reciprocal movement toward controls after ketamine (Figure 4B(ii,iv)). This supports the view that IR can quantify treatment-induced state shifts even when the symptom-level response differs across patients.

### 4.3. Recovery Motifs Are Definable in Both Groups (Figure 4C)

Directional recovery motifs (reverse V-shaped and V-shaped recovery) can be defined in each clinical group using the same logic as in the HKT case study (Figure 4C). The counts are presented to summarize motif definitions and do not imply that non-responders necessarily exhibit fewer recovered loci; rather, they demonstrate that IR-based recovery motifs are definable in both responders and non-responders, consistent with IR functioning as a pharmacodynamic layer beyond binary clinical outcomes.

### 4.4. An Extreme Outlier (PB100) Illustrates Why DEG Is More Fragile than IR in Blood (Figure 4D,E)

Figure 4D highlights a concrete example of biological extremes: a single non-responder (PB100) shows unusually strong up-regulation of 47 genes (z-score > 3 in both PRE and POST), producing a distinct expression-state signature on the heatmap. Enrichment analysis of these 47 genes indicates dominant viral/innate-immune biology (Figure 4E), consistent with an infection-like or strongly innate-activated state. This example also clarifies why outlier handling can have asymmetric effects across layers: excluding PB100 substantially alters DEG overlaps, whereas IR-based overlaps (IncIR/DecIR) remain largely stable (Figure 4F). Together, Figure 4 supports a layered interpretation in which IR preferentially reports upstream state changes that remain informative under heterogeneity, while DEG outputs are more readily dominated by extreme biological states.

### 4.5. Implication: IR Enables Pharmacodynamic Profiling Beyond Symptom-Threshold Crossing

Taken together, the ketamine dataset illustrates a practical separation between molecular engagement and symptom outcomes. Pre-treatment IR signatures in non-responders point to an immune/viral-load state that may constrain benefit (Figure 4A), while IR dynamics still capture treatment engagement and definable recovery motifs in both groups (Figure 4B,C). This reinforces the translational utility of IR as a state-variable layer for monitoring drug response and recovery trajectories in vivo, particularly in blood where downstream expression outputs are sensitive to heterogeneity and outliers (Figure 4D,E).

### 4.6. Interpreting “Responders” and “Non-Responders”: State-Dependent Versus Trait-Dependent Non-Response

In the ketamine dataset discussed here, we interpret non-response primarily as state-dependent: pre-treatment IR signatures in clinically defined non-responders are dominated by viral/innate-immune programs (including the PB100 extreme), consistent with an infection-like immune-load state that may blunt clinical benefit despite detectable molecular engagement. However, responder/non-responder labels should not be assumed to reflect the same mechanism across a variety of interventions. For some therapies, variability in response is influenced by trait-like factors, including pharmacogenomic variation. For example, inter-individual differences in platelet inhibition by aspirin (“aspirin resistance” or hyporesponsiveness) have been linked to genetic variants in COX-1/PTGS1 and related pathways, although clinical and biochemical factors also contribute [57,58,59,60,61]. Taken together, the most informative interpretation of responder/non-responder categories is likely intervention-specific, and IR-based state variables provide a practical way to distinguish state-driven non-response (e.g., high inflammatory/immune load) from trait-driven limitations. In the context of anti-inflammatory interventions, including hangekobokuto (HKT), a state-dependent immune-load model may often be plausible but requires prospective validation across drugs and cohorts with parallel measurement of IR and physiological markers.

## 5. Outliers Are Not Always “Noise”: General Guidance for IR-Centered Biomarker Analyses

Real-world blood transcriptomes inevitably contain outliers. While some reflect technical failures, others represent biologically meaningful extremes (e.g., infection-like innate activation), as exemplified by PB100 in Figure 4D–F. Importantly, outlier handling can markedly reshuffle DEG-based interpretations, whereas IR recovery motifs/modules often remain directionally interpretable with or without such samples, supporting IR as a robust pharmacodynamic readout in heterogeneous clinical cohorts. This motivates a pragmatic approach for IR-centered biomarker analyses [13]:

1. Remove clear technical failures (mapping/QC anomalies and batch artifacts).
2. Do not automatically discard biological extremes; treat them as potentially informative heterogeneity.
3. Report sensitivity analyses (with and without outliers) and prioritize readouts that remain interpretable under both settings.
4. Use IR modules/motifs to interpret outliers, rather than assuming “outlier = noise”, a premise often inherited from DEG-centric workflows.

## 6. Beyond Depression: Why a Homeostatic State Variable Should Generalize to Other Disorders (Including MCI)

Although the evidence base reviewed here is depression-focused, the underlying logic is inherently transdiagnostic: many disorders characterized by chronic stress load, immune–metabolic imbalance, and delayed recovery should manifest measurable deviations in homeostatic regulation. If IR functions as an upstream throttle on effective gene output, it provides a general and mechanistically interpretable biomarker layer for tracking disease burden and therapeutic normalization across conditions.

**Figure 5:**
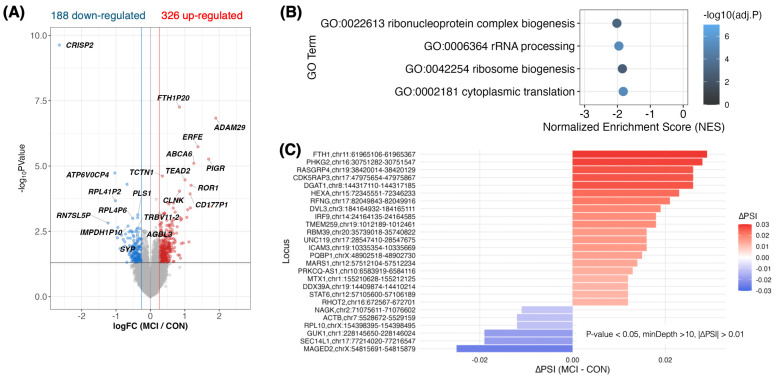
Emory Vascular whole-blood RNA-seq MCI cohort: IR signals were robustly detectable, although they were subtle, motivating longitudinal pharmacodynamic use of IR/DI. (**A**) Volcano plot for MCI (*n* = 99) versus cognitively normal controls (*n* = 101): 514 DEGs (326 up, 188 down) at nominal *p* < 0.05 and |FC| > 1.2. (**B**) GSEA of ranked DEGs highlights significantly repressed gene sets related to ribosome/translation (e.g., ribosome biogenesis, ribonucleoprotein complex biogenesis, cytoplasmic translation, and rRNA processing; negative NES with adjusted *p*-values). (**C**) IR events detected under a lenient cutoff (nominal *p* < 0.05, minCov > 10, |ΔIR| > 0.01): 20 IncIR and 6 DecIR loci (typically ~1–3% ΔIR); the bar plot shows top IncIR events (largest ΔIR) and all DecIR events. Data source: Emory Vascular study via the AD Knowledge Portal [62]. Source/license: panels (**A**–**C**) were newly created for this review. Abbreviations: CN, cognitively normal controls; MCI, mild cognitive impairment; DEG, differentially expressed gene; GSEA, gene set enrichment analysis; NES, normalized enrichment score; IR, intron retention; IncIR, increased intron retention; DecIR, decreased intron retention; ΔIR, difference in IR ratio (MCI minus CN); PD, pharmacodynamic.

A particularly stringent test case is early cognitive decline, where sensitive biomarkers are required at the MCI stage to detect risk, monitor progression, and quantify intervention effects before irreversible damage accrues. Critically, MCI is also a setting where peripheral blood is unlikely to directly mirror the primary CNS drivers, making it an ideal stress test for whether IR remains useful when the sampled tissue is biologically distal. As an illustrative example, we performed a side-by-side DEG and IR re-analysis of a whole-blood RNA-seq MCI cohort (Emory Vascular study; MCI *n* = 99 vs. cognitively normal controls, CN *n* = 101) (Figure 5) [62]. In this cohort, DEG analysis produced a strong cross-sectional contrast (>500 DEGs) with enrichment pointing to coordinated repression of ribosome/translation-related programs, consistent with large-scale Japanese blood transcriptome studies reporting early shifts in ribosome/translation modules at the CN→MCI transition [63,64]. By contrast, IR differences were fewer and smaller in amplitude, as expected when blood acts as an indirect reporter rather than the primary site of pathology. However, IR signals were nonetheless detectable when statistical power was adequate, yielding a small set of significant IR loci with subtle effect sizes (typically ~1–3% ΔIR) (Figure 5C).

Altogether, this example helps define a practical boundary condition for translational use. Depression—where peripheral inflammation can be proximal to pathophysiology—can yield larger and more numerous blood IR signals even in smaller cohorts, whereas MCI—where blood is biologically distal—may show weaker cross-sectional IR effects that become reliably measurable only in adequately powered cohorts. Importantly, the value proposition of IR/DI in such settings may be greatest in longitudinal, within-individual monitoring, where IR trajectories can quantify treatment engagement and recovery kinetics even when baseline case–control separation is modest. Systematic replication across independent MCI cohorts, ideally coupled to longitudinal designs and modifiable risk dimensions (e.g., sleep/circadian disruption, vascular–immune load, and intervention response), is therefore a key next step and provides a natural path for an independent, dedicated study.

## 7. Concluding Perspective

The central message emerging from the HKT and ketamine case studies is that IR behaves like a controllable homeostatic state variable: it deviates under disease/stress load, it normalizes with effective intervention, it stratifies response heterogeneity, and it can reveal plausible mechanistic axes (notably inflammation/innate-immune activation) that help interpret non-response. These properties position IR as an unusually practical biomarker layer for drug response and recovery monitoring in depression, complementing conventional DEG readouts that often reflect context-sensitive downstream abundance outputs.

Several pragmatic design principles follow. First, directionality matters: recovery motifs such as V-shaped and reverse V-shaped IR trajectories provide interpretable pharmacodynamic “dials” that can quantify normalization. Second, robustness matters: IR programs show stronger cross-cohort reproducibility than DEG lists in whole blood and can remain directionally interpretable even in the presence of biological outliers, supporting real-world deployment. Third, translation is tractable: genome-wide discovery can be followed by targeted clinical assays measuring a small panel of sentinel IR/DI events (e.g., junction-/intron-specific RT-qPCR, ddPCR, or targeted amplicon sequencing), provided that pre-analytics, event-level normalization, and cell-composition effects are handled explicitly.

Finally, while this review emphasizes depression as a proof of concept, the same pharmacodynamic logic should generalize to other disorders in which homeostatic dysregulation is central. Early cognitive decline (MCI) provides a stringent “distal-tissue” test case: cross-sectional IR/DI effects in blood may be modest, yet reproducible shifts can emerge in adequately powered cohorts, and the greatest clinical value is likely to come from longitudinal trajectories that track intervention engagement and recovery rather than baseline case–control separation alone. Establishing which IR/DI events are reusable across independent MCI cohorts and whether they track clinically meaningful trajectories is therefore a clear next step and an appropriate focus for a dedicated follow-up study.

## Figures and Tables

**Table 1:** Mechanistic literature supporting hangekobokuto/BHT and correspondence to recovered IR genes.

Ref.	Formula/Model	Key Reported Mechanism (Very Short)	Corresponding Recovered IR Genes (Motif; Tag)
Endo M et al. (J Smooth Muscle Res. 2022;58:78–88) [31]	HKT; POI model	↓ neutrophil/macrophage infiltration; ↓ iNOS; ↓ NF-κB; ↑ NGF	NOSIP (V; NO axis); CXCL2 (rev-V; chemokine recruitment); FAS (rev-V; death signaling)
Mihara T et al. (Inflammation. 2017;40(4):1331–1341) [34]	Honokiol (Magnolia component); inflammation/POI context	↓ cytokines; ↓ iNOS	NOSIP (V; NO axis); CXCL2 (rev-V; chemokine recruitment); TRIM16 (rev-V; inflammasome control)
Liu L et al. (J Cell Mol Med. 2023;27:3339–3353) [32]	BHT/BXHPD; CUMS depression	↓ IL-6/TNF-α/IL-1β; ↑ IL-10/IL-4; ↓ microglia activation; ↑ M2 polarization	IL17RB (rev-V; IL-17 axis); NFATC4 (V; immune TF); OAS2 (V; innate antiviral); TRIM16 (rev-V; inflammasome control)
Jia KK et al. (J Ethnopharmacol. 2017;209:219–229) [33]	BHT/BXHPD; CUMS + metabolic/inflammasome	↓ NLRP3 inflammasome activation; improved metabolic signaling	TRIM16 (rev-V; inflammasome control); ERLIN1 (V; ER homeostasis); CERT1 (rev-V; ceramide transport)
Yang HN et al. (J Ethnopharmacol. 2026;359:121024) [35]	BXHPD; OGT–CTSB–NLRP3 axis	↓ OGT/CTSB O-GlcNAc; ↓ ROS/LMP; ↓ NLRP3 activation	ALG5 (rev-V; N-glycan); GMPPA (rev-V; N-glycan); RGP1 (rev-V; Golgi trafficking); TVP23C (rev-V; Golgi trafficking); AP2M1 (V; clathrin endocytosis)
Kwon HJ et al. (Tradit Med Res. 2025;10(5):26) [36]	BHT; meta-analysis/network pharmacology	Neuroinflammation emphasis; IL-17 signaling suggested	IL17RB (rev-V; IL-17 axis); NOSIP (V; NO axis); CXCL2 (rev-V; chemokine recruitment); OAS2 (V; innate antiviral); TRIM16 (rev-V; inflammasome control)

The symbols ↑ and ↓ represent upregulation and downregulation.

**Table 2:** DEG–IR cross-walk linking IR-defined recovery axes (state layer) to recovered DEG outputs (output layer) in the HKT cohort. Representative IR nodes are listed for each axis; recovery patterns (V-shape, reverse V-shape) follow the definitions in Figure 2B,C,E.

Recovered DEG (Output)	Recovery Pattern	Axis Label	Representative Recovered IR Nodes (State)	Network Evidence (STRING/Cytoscape)
G0S2	reverse V-shape	Immune/innate	CXCL2 (rev-V), OAS2 (V), IL17RB (rev-V), TRIM16 (rev-V), NFATC4 (V), FAS (rev-V), NOSIP (V)	STRING ≥ 0.4: CXCL2-centered neighborhood connects to G0S2 and OAS2 (Figure 3A)
DOCK6	reverse V-shape	Cytoskeleton/adhesion	EOGT (rev-V), MYH10 (rev-V), MYLK (rev-V), LIMS2 (rev-V), CELSR2 (rev-V)	STRING ≥ 0.4: DOCK6–EOGT link (Figure 3A)
UTS2B	reverse V-shape	Peptide signaling/other	—	No stable DEG–IR edge at STRING ≥ 0.4
TUB	reverse V-shape	Cilia/ciliary trafficking	AHI1 (V), CEP104 (V), NPHP1 (V), CCDC24 (V), DNHD1 (V)	Axis-level alignment; no highlighted DEG–IR edge at STRING ≥ 0.4
ENSG00000264187	reverse V-shape	Unannotated/non-coding (ID)	—	No stable DEG–IR edge at STRING ≥ 0.4
ELL2	reverse V-shape	Transcription/RNA-processing	DDX5 (V), PRMT7 (V), SMARCD2 (V), KMT5B (V), SMC4 (V), ZWINT (V)	Axis-level alignment; no highlighted DEG–IR edge at STRING ≥ 0.4
TXNDC5	reverse V-shape	ER/proteostasis	ERLIN1 (V), MFN2 (V), SPG7 (V), NDUFA5 (V), FOXRED1 (V), TEFM (rev-V), SIGMAR1 (V)	Axis-level alignment; no highlighted DEG–IR edge at STRING ≥ 0.4
BHLHE41	reverse V-shape	Immune/innate-associated TF	NFATC4 (V), CXCL2 (rev-V), IL17RB (rev-V)	Axis-level alignment; no highlighted DEG–IR edge at STRING ≥ 0.4
ABCB9	reverse V-shape	Endomembrane/lysosome/trafficking	AP2M1 (V), GBF1 (rev-V), ALG5 (rev-V), GMPPA (rev-V), RGP1 (rev-V), TVP23C (rev-V), CERT1 (rev-V)	Axis-level alignment; no highlighted DEG–IR edge at STRING ≥ 0.4
NPIPB6	reverse V-shape	Unannotated/unclear	—	No stable DEG–IR edge at STRING ≥ 0.4
SMIM11	V-shape	Hematopoiesis/composition	—	No stable DEG–IR edge at STRING ≥ 0.4
C11orf16	V-shape	Hematopoiesis/composition	—	No stable DEG–IR edge at STRING ≥ 0.4
PHACTR3	V-shape	Cytoskeleton/actin regulation	MYH10 (rev-V), MYLK (rev-V), LIMS2 (rev-V)	Axis-level alignment; no highlighted DEG–IR edge at STRING ≥ 0.4
ALPK3	V-shape	Cytoskeleton/contractile signaling	MYH10 (rev-V), MYLK (rev-V)	Axis-level alignment; no highlighted DEG–IR edge at STRING ≥ 0.4
DLGAP2	V-shape	Other/unclear	—	No stable DEG–IR edge at STRING ≥ 0.4
ZNF625	V-shape	Chromatin/transcription	BRD9 (rev-V), KMT5B (V), ZNF714 (rev-V), ZNF789 (rev-V)	Axis-level alignment; no highlighted DEG–IR edge at STRING ≥ 0.4
ENSG00000261341	V-shape	Unannotated/non-coding (ID)	—	No stable DEG–IR edge at STRING ≥ 0.4

## Data Availability

The raw RNA-seq datasets used in this study can be downloaded from the DDBJ Sequence Read Archive under accession numbers DRR540207–DRR540228, which are linked to the BioProject accession number PRJDB17815 [20] the NCBI Gene Expression Omnibus (GEO) under accession number GSE185855 [21], and https://www.synapse.org/Synapse:syn18909507 (accessed on 27 October 2020) (Emory Vascular study).

## Supplementary Materials

The following supporting information can be downloaded at: https://www.mdpi.com/article/10.3390/ijms27083539/s1.

## References

[1] Okada N., Maruko A., Oshima K., Nishi A., Kobayashi Y. (2026). The IR-Homeostat Hypothesis: Intron Retention as an Evolutionarily Conserved Fine-Tuning Layer and a Reversible Blood Biomarker of Homeostatic Dysregulation in Mood Disorders. Int. J. Mol. Sci..

[2] World Health Organization (2017). Depression and Other Common Mental Disorders: Global Health Estimates.

[3] Howren M.B., Lamkin D.M., Suls J. (2009). Associations of depression with C-reactive protein, IL-1, and IL-6: A meta-analysis. Psychosom. Med..

[4] Dowlati Y., Herrmann N., Swardfager W., Liu H., Sham L., Reim E.K., Lanctôt K.L. (2010). A meta-analysis of cytokines in major depression. Biol. Psychiatry.

[5] Haapakoski R., Mathieu J., Ebmeier K.P., Alenius H., Kivimaki M. (2015). Cumulative meta-analysis of interleukins 6 and 1beta, tumour necrosis factor alpha and C-reactive protein in patients with major depressive disorder. Brain Behav. Immun..

[6] Bullmore E. (2018). The Inflamed Mind: A Radical New Approach to Depression.

[7] Khandaker G.M., Pearson R.M., Zammit S., Lewis G., Jones P.B. (2014). Association of serum interleukin 6 and C-reactive protein in childhood with depression and psychosis in young adult life: A population-based longitudinal study. JAMA Psychiatry.

[8] Prather A.A., Rabinovitz M., Pollock B.G., Lotrich F.E. (2009). Cytokine-induced depression during IFN-alpha treatment: The role of IL-6 and sleep quality. Brain Behav. Immun..

[9] Raison C.L., Rutherford R.E., Woolwine B.J., Shuo C., Schettler P., Drake D.F., Haroon E., Miller A.H. (2013). A randomized controlled trial of the tumor necrosis factor antagonist infliximab for treatment-resistant depression: The role of baseline inflammatory biomarkers. JAMA Psychiatry.

[10] Hori H., Sasayama D., Teraishi T., Yamamoto N., Nakamura S., Ota M., Hattori K., Kim Y., Higuchi T., Kunugi H. (2016). Blood-based gene expression signatures of medication-free outpatients with major depressive disorder: Integrative genome-wide and candidate gene analyses. Sci. Rep..

[11] Begley C.G., Ellis L.M. (2012). Drug development: Raise standards for preclinical cancer research. Nature.

[12] Baker M. (2016). 1,500 scientists lift the lid on reproducibility. Nature.

[13] Leek J.T., Scharpf R.B., Bravo H.C., Simcha D., Langmead B., Johnson W.E., Geman D., Baggerly K., Irizarry R.A. (2010). Tackling the widespread and critical impact of batch effects in high-throughput data. Nat. Rev. Genet..

[14] Jaffe A.E., Irizarry R.A. (2014). Accounting for cellular heterogeneity is critical in epigenome-wide association studies. Genome Biol..

[15] Houseman E.A., Accomando W.P., Koestler D.C., Christensen B.C., Marsit C.J., Nelson H.H., Wiencke J.K., Kelsey K.T. (2012). DNA methylation arrays as surrogate measures of cell mixture distribution. BMC Bioinform..

[16] Okada N., Oshima K., Iwasaki Y., Maruko A., Matsumura K., Iioka E., Vu T.-D., Fujitsuka N., Nishi A., Sugiyama A. (2021). Intron retention as a new pre-symptomatic marker of aging and its recovery to the normal state by a traditional Japanese multi-herbal medicine. Gene.

[17] Vu T.D., Ito N., Oshima K., Maruko A., Nishi A., Mizoguchi K., Odaguchi H., Kobayashi Y., Okada N. (2022). Intron retention is a stress response in sensor genes and is restored by Japanese herbal medicines: A basis for future clinical applications. Gene.

[18] Wong J.J.L., Schmitz U. (2022). Intron retention: Importance, challenges, and opportunities. Trends Genet..

[19] Monteuuis G., Wong J.J.L., Bailey C.G., Schmitz U., Rasko J.E.J. (2019). The changing paradigm of intron retention: Regulation, ramifications and recipes. Nucleic Acids Res..

[20] Okada N., Oshima K., Maruko A., Sekine M., Ito N., Wakasugi A., Mori E., Odaguchi H., Kobayashi Y. (2024). Intron retention as an excellent marker for diagnosing depression and for discovering new potential pathways for drug intervention. Front. Psychiatry.

[21] Okada N., Oshima K., Maruko A., Kobayashi Y. (2025). Intron retention: A novel method for evaluating the response to ketamine in patients with treatment-resistant depression. npj Ment. Health Res..

[22] Boutz P.L., Bhutkar A., Sharp P.A. (2015). Detained introns are a novel, widespread class of post-transcriptionally spliced introns. Genes Dev..

[23] Mauger O., Lemoine F., Scheiffele P. (2016). Targeted intron retention and excision for rapid gene regulation in response to neuronal activity. Neuron.

[24] Tan Z.-W., Fei G., A Paulo J., Bellaousov S., Martin S.E.S., Duveau D.Y., Thomas C.J., Gygi S.P., Boutz P.L., Walker S. (2020). O-GlcNAc regulates gene expression by controlling detained intron splicing. Nucleic Acids Res..

[25] Wong J.J.-L., Ritchie W., Ebner O.A., Selbach M., Wong J.W., Huang Y., Gao D., Pinello N., Gonzalez M., Baidya K. (2013). Orchestrated intron retention regulates normal granulocyte differentiation. Cell.

[26] Pimentel H., Parra M., Gee S.L., Mohandas N., Pachter L., Conboy J.G. (2016). A dynamic intron retention program enriched in RNA processing genes regulates gene expression during terminal erythropoiesis. Nucleic Acids Res..

[27] Naro C., Jolly A., Di Persio S., Bielli P., Setterblad N., Alberdi A.J., Vicini E., Geremia R., De la Grange P., Sette C. (2017). An orchestrated intron retention program in meiosis controls timely usage of transcripts during germ cell differentiation. Dev. Cell.

[28] Ullrich S., Guigó R. (2020). Dynamic changes in intron retention are tightly associated with regulation of splicing factors and proliferative activity during B-cell development. Nucleic Acids Res..

[29] Cathomas F., Bevilacqua L., Ramakrishnan A., Kronman H., Costi S., Schneider M., Chan K.L., Li L., Nestler E.J., Shen L. (2022). Whole blood transcriptional signatures associated with rapid antidepressant response to ketamine in patients with treatment resistant depression. Transl. Psychiatry.

[30] Zhang D., Ji Y., Chen X., Chen R., Wei Y., Peng Q., Lin J., Yin J., Li H., Cui L. (2022). Peripheral blood circular RNAs as a biomarker for major depressive disorder and prediction of possible pathways. Front. Neurosci..

[31] Endo M., Oikawa T., Tonooka M., Hanawa T., Odaguchi H., Hori M. (2022). Hangekobokuto, a traditional Japanese herbal medicine, ameliorates postoperative ileus through its anti-inflammatory action. J. Smooth Muscle Res..

[32] Liu L., Zhang R., Chen C., Xia C., Yao G., He X., Xia B. (2023). The effect of Banxia-houpo decoction on CUMS-induced depression by promoting M2 microglia polarization via TrkA/Akt signalling. J. Cell Mol. Med..

[33] Jia K.K., Zheng Y.J., Zhang Y.X., Liu J.H., Jiao R.Q., Pan Y., Kong L.D. (2017). Banxia-houpu decoction restores glucose intolerance in CUMS rats through improvement of insulin signaling and suppression of NLRP3 inflammasome activation in liver and brain. J. Ethnopharmacol..

[34] Mihara T., Mikawa S., Kaji N., Endo M., Oikawa T., Jan T.R., Ozaki H., Hori M. (2017). Therapeutic action of honokiol on postoperative ileus via downregulation of iNOS gene expression. Inflammation.

[35] Yang H.N., Peng Q., Shuang R., Guo Z., Yang H., Chen C., Tao W., Liu L. (2026). Banxia Houpo Decoction reduces lysosomal leakage of prefrontal astrocytes through the OGT-CTSB-NLRP3 pathway to improve depressive-like behaviors. J. Ethnopharmacol..

[36] Kwon H.J., Seung H.B., Tran K.N., Yang I.J., Kim S.H. (2025). Efficacy and mechanism of Chinese herbal medicine Banxia-Houpo-Tang for depression: A meta-analysis and network pharmacology analysis. Tradit. Med. Res..

[37] De Brito O.M., Scorrano L. (2008). Mitofusin 2 tethers endoplasmic reticulum to mitochondria. Nature.

[38] Gao X., Bonzerato C.G., Wojcikiewicz R.J.H. (2022). Binding of the erlin1/2 complex to the third intralumenal loop of IP3R1 triggers its ubiquitin-proteasomal degradation. J. Biol. Chem..

[39] Fassone E., Duncan A.J., Taanman J.-W., Pagnamenta A.T., Sadowski M.I., Holand T., Qasim W., Rutland P., Calvo S.E., Mootha V.K. (2010). FOXRED1, encoding an FAD-dependent oxidoreductase complex-I-specific molecular chaperone, is mutated in infantile-onset mitochondrial encephalopathy. Hum. Mol. Genet..

[40] Machida Y.J., Machida Y., Chen Y., Gurtan A.M., Kupfer G.M., D’ANdrea A.D., Dutta A. (2006). UBE2T is the E2 in the Fanconi anemia pathway and undergoes negative autoregulation. Mol. Cell..

[41] Seo D.W., You S.Y., Chung W.-J., Cho D.-H., Kim J.-S., Oh J.S. (2015). Zwint-1 is required for spindle assembly checkpoint function and kinetochore-microtubule attachment during oocyte meiosis. Sci Rep..

[42] Freeman L., Aragon-Alcaide L., Strunnikov A. (2000). The condensin complex governs chromosome condensation and mitotic transmission of rDNA. J. Cell Biol..

[43] Huis In’t Veld P.J., Jeganathan S., Petrovic A., Singh P., John J., Krenn V., Weissmann F., Bange T., Musacchio A. (2016). Molecular basis of outer kinetochore assembly on CENP-T. eLife.

[44] Hsiao Y.-C., Tong Z.J., Westfall J.E., Ault J.G., Page-McCaw P.S., Ferland R.J. (2009). Ahi1, whose human ortholog is mutated in Joubert syndrome, is required for Rab8a localization, ciliogenesis and vesicle trafficking. Hum. Mol. Genet..

[45] Frikstad K.-A.M., Molinari E., Thoresen M., Ramsbottom S.A., Hughes F., Letteboer S.J., Gilani S., Schink K.O., Stokke T., Geimer S. (2019). A CEP104-CSPP1 complex is required for formation of primary cilia competent in Hedgehog signaling. Cell Rep..

[46] Hildebrandt F., Zhou W. (2007). Nephronophthisis-associated ciliopathies. J. Am. Soc. Nephrol..

[47] Shannon P., Markiel A., Ozier O., Baliga N.S., Wang J.T., Ramage D., Amin N., Schwikowski B., Ideker T. (2003). Cytoscape: A software environment for integrated models of biomolecular interaction networks. Genome Res..

[48] Szklarczyk D., Gable A.L., Nastou K.C., Lyon D., Kirsch R., Pyysalo S., Doncheva N.T., Legeay M., Fang T., Bork P. (2021). The STRING database in 2021: Customizable protein-protein networks, and functional characterization of user-uploaded gene/measurement sets. Nucleic Acids Res..

[49] Capucetti A., Albano F., Bonecchi R. (2020). Multiple roles for chemokines in neutrophil biology. Front. Immunol..

[50] Schwartz S.L., Conn G.L. (2019). RNA regulation of the antiviral protein 2′-5′-oligoadenylate synthetase. Wiley Interdiscip. Rev. RNA.

[51] Miyamoto Y., Yamauchi J., Sanbe A., Tanoue A. (2007). Dock6, a Dock-C subfamily guanine nucleotide exchanger, has the dual specificity for Rac1 and Cdc42 and regulates neurite outgrowth. Exp. Cell Res..

[52] Müller R., Jenny A., Stanley P. (2013). The EGF Repeat-Specific O-GlcNAc-Transferase Eogt Interacts with Notch Signaling and Pyrimidine Metabolism Pathways in Drosophila. PLoS ONE.

[53] Okada N., Oshima K., Maruko A., Miki R., Iwasaki Y., Kobayashi Y. (2026). Intron retention resolves microgravity and non-gravitational stress programs across immune organs in spaceflight. arXiv.

[54] Park D., Han C.Z., Elliott M.R., Kinchen J.M., Trampont P.C., Das S., Collins S., Lysiak J.J., Hoehn K.L., Ravichandran K.S. (2011). Continued clearance of apoptotic cells critically depends on the phagocyte Ucp2 protein. Nature.

[55] Klein L., Kyewski B., Allen P.M., Hogquist K.A. (2014). Positive and negative selection of the T cell repertoire: What thymocytes see (and don’t see). Nat. Rev. Immunol..

[56] Zhou T.-A., Hsu H.-P., Tu Y.-H., Cheng H.-K., Lin C.-Y., Chen N.-J., Tsai J.-W., A Robey E., Huang H.-C., Hsu C.-L. (2022). Thymic macrophages consist of two populations with distinct localization and origin. eLife.

[57] Freedman J.E. (2006). The aspirin resistance controversy: Clinical entity or platelet heterogeneity?. Circulation.

[58] Fitzgerald R., Pirmohamed M. (2011). Aspirin resistance: Effect of clinical, biochemical and genetic factors. Pharmacol. Ther..

[59] Da Silva G.F., Lopes B.M., Moser V., Ferreira L.E. (2023). Impact of pharmacogenetics on aspirin resistance: A systematic review. Arq. Neuropsiquiatr..

[60] Maree A.O., Curtin R.J., Chubb A., Dolan C., Cox D., O’BRien J., Crean P., Shields D.C., Fitzgerald D.J. (2005). Cyclooxygenase-1 haplotype modulates platelet response to aspirin. J. Thromb. Haemost..

[61] Li C.-X., Sun L.-C., Wang Y.-Q., Liu T.-T., Cai J.-R., Liu H., Ren Z., Yi Z. (2024). The associations of candidate gene polymorphisms with aspirin resistance in patients with ischemic disease: A meta-analysis. Hum. Genom..

[62] AD Knowledge Portal The Emory_Vascular Study (Emory_Vascular; syn18909507). https://adknowledgeportal.synapse.org/Explore/Studies/DetailsPage/StudyDetails?Study=syn18909507.

[63] Yamakawa A., Suganuma M., Mitsumori R., Niida S., Ozaki K., Shigemizu D. (2025). Alzheimer’s disease may develop from changes in the immune system, cell cycle, and protein processing following alterations in ribosome function. Sci. Rep..

[64] Shigemizu D., Mori T., Akiyama S., Higaki S., Watanabe H., Sakurai T., Niida S., Ozaki K. (2020). Identification of potential blood biomarkers for early diagnosis of Alzheimer’s disease through RNA sequencing analysis. Alzheimers Res. Ther..

