# Supplementary material for: Intron Retention as a Homeostatic State Variable for Drug Response and Recovery: Lessons from Depression for Broader Applications
Source: Int J Mol Sci. 2026 Apr 16;27(8):3539. doi: 10.3390/ijms27083539 (PMC13115884; doi:10.3390/ijms27083539)
Supplement: Supplementary file 1 [file ijms-27-03539-s001.zip › Table S1.pdf]

Table S1 Updated classification of 64 protein-coding recovery IR genes

Category	n / 64	%	Genes
Inflammation	21	32.8	ADCY4, BRD9, CDCA3, CERT1, CXCL2, DDX5, ERLIN1, FAS, IL17RB, MYLK, NCSTN, NDRG2, NFATC4, NOSIP, OAS2, PRMT7, ROBO3, SLC22A5, TRIM16, UBE2T, USP21
Cilia	8	12.5	AHI1, C21orf58, CELSR2, CEP104, DNHD1, GBF1, NAPEPLD, NPHP1
Mitochondria	7	10.9	FOXRED1, MFN2, MYH10, NDUFA5, SIGMAR1, SPG7, TEFM
Hematopoiesis	3	4.7	CDIN1, EOGT, SMARCD2
DNA repair / Recombination	2	3.1	PMS2, REC8
Genome integrity (chromatin/kinetochore/condensin)	4	6.3	CENPT, KMT5B, SMC4, ZWINT
Ciliogenesis-support (Golgi/sugar chain/trafficking/centrosome-adjacent)	8	12.5	ALG5, AP2M1, CCHCR1, FAM153A, GMPPA, RGP1, TMEM25, TVP23C
Antiviral / innate-immune	2	3.1	POLR3A, SKIC3
Residual Others (unassigned / multi-functional)	9	14.1	ABHD14A-ACY1, ATXN7L2, BCKDK, CCDC24, FAM131B, LIMS2, PCSK4, ZNF714, ZNF789
